# Supplementary material for: Population codes of prior knowledge learned through environmental regularities
Source: Sci Rep. 2021 Jan 12;11:640. doi: 10.1038/s41598-020-79366-z (PMC7804143; doi:10.1038/s41598-020-79366-z)
Supplement: Supplementary file 1 — Supplementary Information. [file 41598_2020_79366_MOESM1_ESM.pdf]

Population codes of prior knowledge learned through environmental regularities

Silvan C. Quax[1,*], Sander E. Bosch[1,2], Marius V. Peelen[1], and Marcel A. J. van Gerven[1]

[1]Donders Institute for Brain, Cognition and Behaviour, Radboud University, Nijmegen, The Netherlands
[2]Vrije Universiteit, Amsterdam, The Netherlands
[*]s.c.quax@gmail.com

Supplementary information

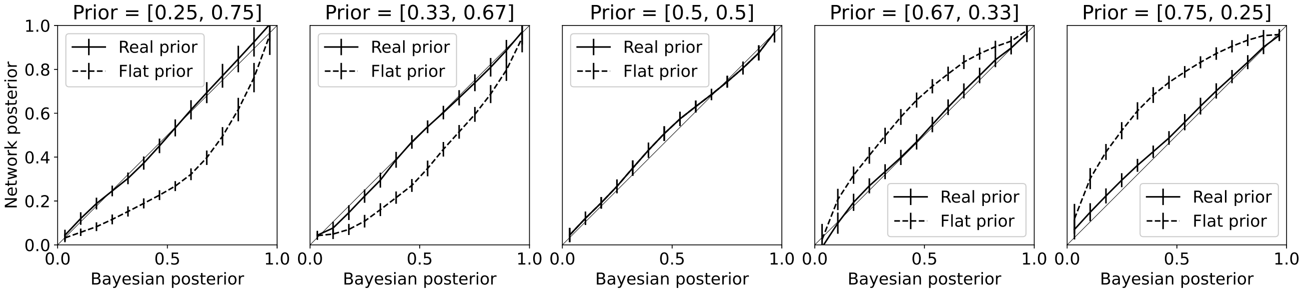

Figure S1. Network posterior matches optimal Bayesian estimates for MSE loss. A neural network optimized with a mean-squared-error loss also learned to choose each class with a certain probability by getting feedback about correct or incorrect classification. Five prior probability ratios were tested: [0.25, 0.75], [0.33,0.67], [0.5, 0.5], [0.67, 0.33] and [0.75, 0.25] for class 1 and class 2 respectively.

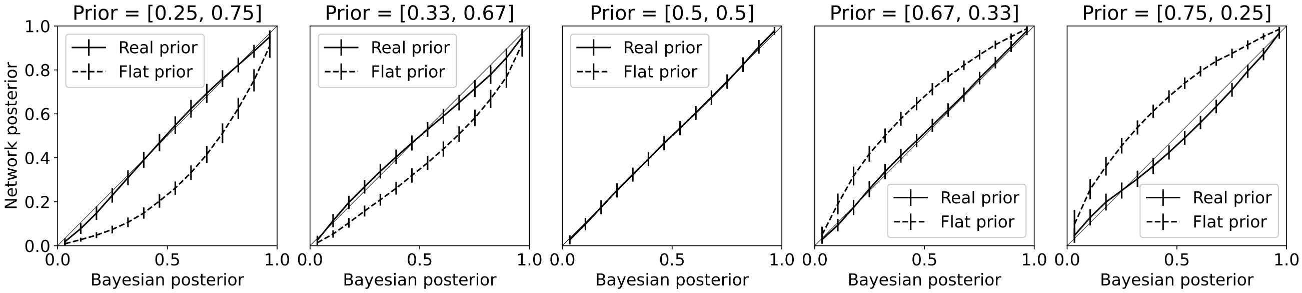

Figure S2. Network posterior matches optimal Bayesian estimates for Leaky-ReLU activation function. A neural network with a Leaky-ReLU activation function also learned to choose each class with a certain probability by getting feedback about correct or incorrect classification. Five prior probability ratios were tested: [0.25, 0.75], [0.33,0.67], [0.5, 0.5], [0.67, 0.33] and [0.75, 0.25] for class 1 and class 2 respectively.

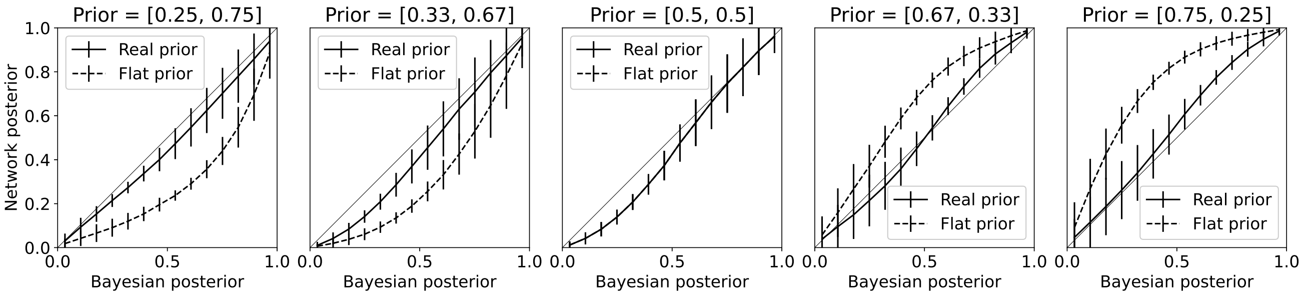

Figure S3. Network posterior matches optimal Bayesian estimates for Sigmoid activation function. A neural network with a Sigmoid activation function also learned to choose each class with a certain probability by getting feedback about correct or incorrect classification, although it had more difficulty to learn the task. The network posteriors matched the Bayesian posterior less well in this network. Five prior probability ratios were tested: [0.25, 0.75], [0.33,0.67], [0.5, 0.5], [0.67, 0.33] and [0.75, 0.25] for class 1 and class 2 respectively.

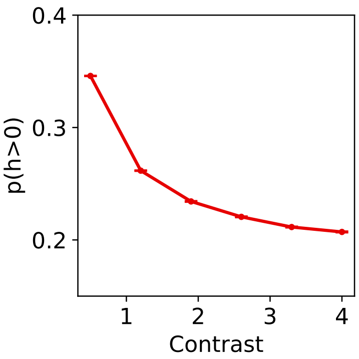

Figure S4. Number of neurons being activated decreases with input contrast. The average sparsity of neural responses is plotted for the different contrast values. Here, no distinction is made depending on whether the input stimulus led to a high posterior probability for one of the classes as in Fig 4A.

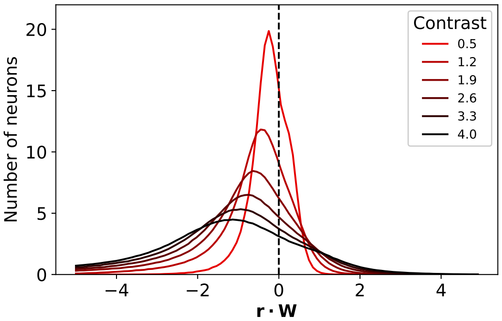

Figure S5. Higher contrast leads to stronger template matches and mismatches. The dot product between the input and the weights for every neuron was calculated to determine the degree of template matching. These were shifted with the threshold (bias) to better visualize when a neuron would become activated. A histogram binning these template matching values in 51 bins for all stimuli was made, showing the average number of neurons with a template matching value. This shows that for low contrast values, all neurons have a low degree of template matching (around 0), with those neurons crossing their activation threshold becoming only weakly activated. For high contrast values on the other hand, there are more clear matches or mismatches, with those neurons crossing their activation threshold becoming more strongly activated.

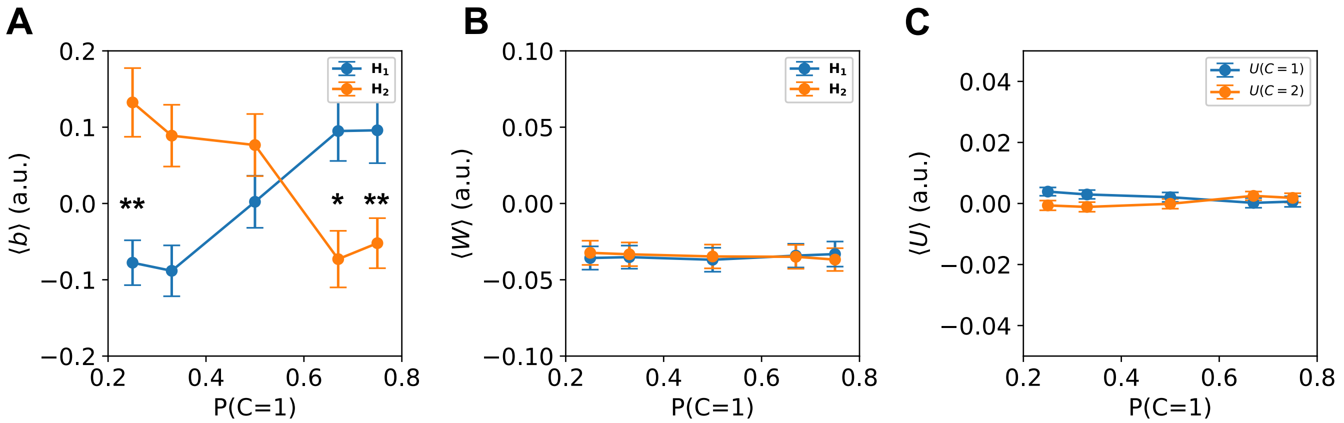

Figure S6. Prior probability encoded by changing activation thresholds with MSE loss. A neural network optimized with a mean-squared-error loss develop the same mechanism as the network optimized through a cross-entropy loss. **(A)** Only the biases of the hidden neurons changed as a result of different prior probabilities, where a higher probability of a class led to higher bias values in neurons coding for this class, thus lowering the activation threshold in these neurons. **(B)** The hidden weights did not change with prior probability. **(C)** Neither did the output weights. Error bars represent standard error over the parameters. (* $0.005 < p < 0.05$, ** $p < 0.005$).

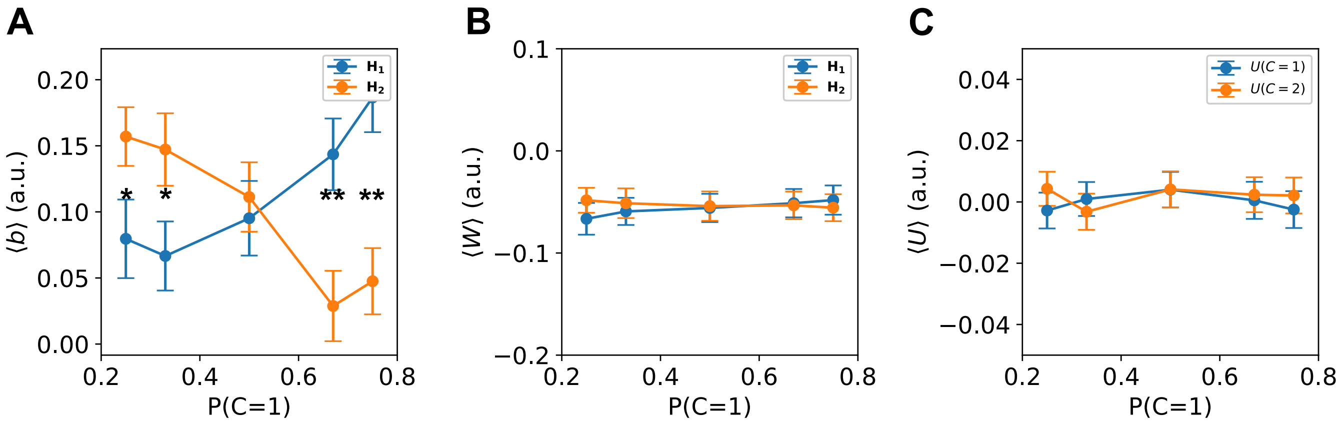

Figure S7. Prior probability encoded by changing activation thresholds with Leaky-ReLU activation function. A neural network optimized with a mean-squared-error loss develop the same mechanism as the network optimized through a cross-entropy loss. **(A)** Only the biases of the hidden neurons changed as a result of different prior probabilities, where a higher probability of a class led to higher bias values in neurons coding for this class, thus lowering the activation threshold in these neurons. **(B)** The hidden weights did not change with prior probability. **(C)** Neither did the output weights. Error bars represent standard error over the parameters. (* $0.005 < p < 0.05$, ** $p < 0.005$).

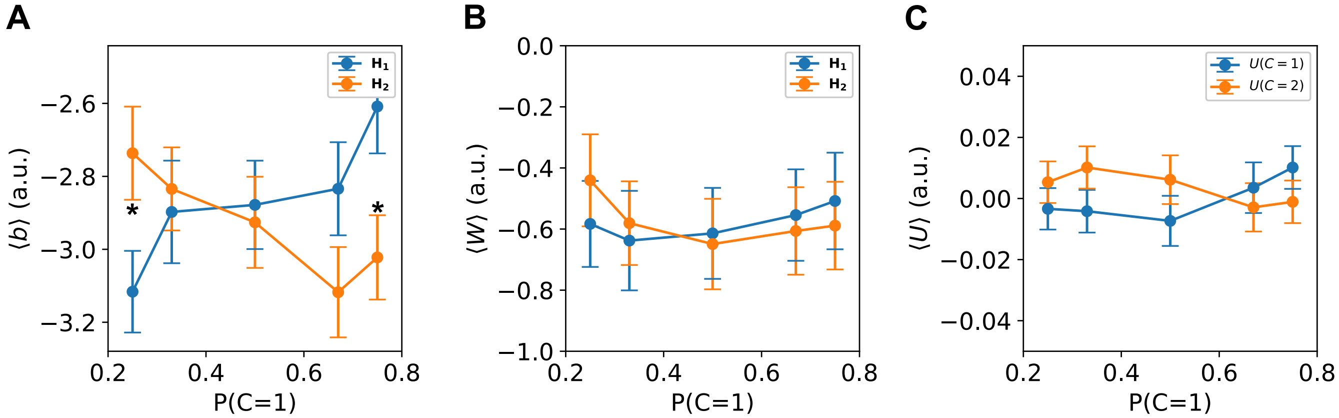

Figure S8. Prior probability encoded by changing activation thresholds with Sigmoid activation function. A neural network optimized with a mean-squared-error loss develop the same mechanism as the network optimized through a cross-entropy loss. **(A)** Only the biases of the hidden neurons changed as a result of different prior probabilities, where a higher probability of a class led to higher bias values in neurons coding for this class, thus lowering the activation threshold in these neurons. **(B)** The hidden weights did not change with prior probability. **(C)** Neither did the output weights. Error bars represent standard error over the parameters. (* $p < 0.05$).

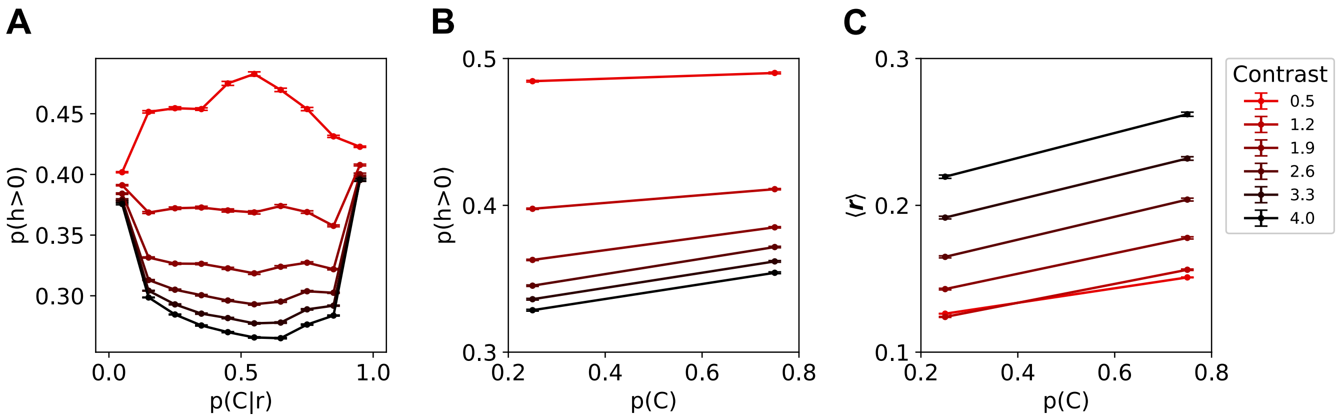

Figure S9. Neural activity changes due to input and cued prior uncertainty. **(A)** Number of neurons with non-zero activity in response to both stimuli from class 1 and class 2 when no prior is cued. Fewer neurons in the hidden layer responded when the input contrast was higher. On the other hand, more neurons responded when the posterior was certain which class was presented. **(B)** Number of neurons with non-zero activity in response to stimuli from class 1 when a prior indicating higher probability for class 1 is cued (p(C)=0.75) and when a prior indicating a lower probability for class 1 is cued (p(C)=0.25). While fewer neurons responded to higher contrast stimuli, more neurons were active when the prior probability of the presented stimulus class was higher. This is the same effect as observed in Fig 4B, although weaker. **(C)** The average activity of the population, in response to stimuli from class 1, is shown when a prior indicating higher probability for class 1 is cued (p(C)=0.75) and when a prior indicating a lower probability for class 1 is cued (p(C)=0.25). The average activity increased both with stimulus contrast and prior probability as in Fig 4C, although the effect is again weaker.

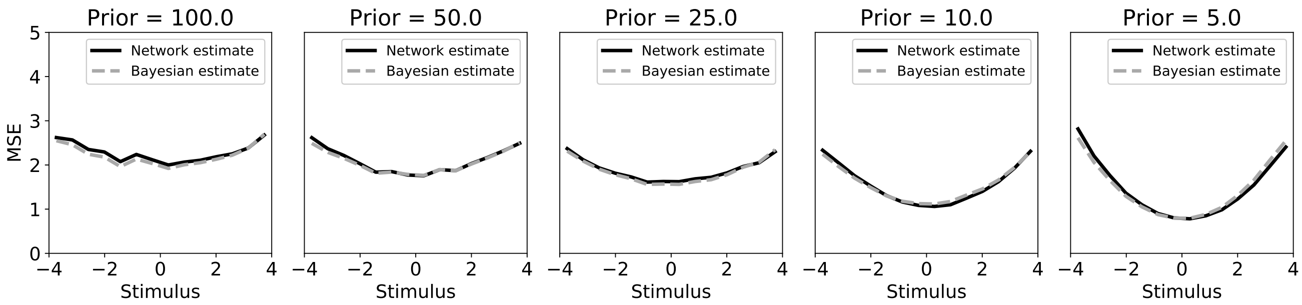

Figure S10. Prior probability lowers estimation error. When a stimulus value has a higher prior probability the error in the estimation produced by the network (solid line) is lower, while stimulus values with low probability result in higher estimation errors. The performance of the network matches the Bayesian estimate (dashed line) very closely (note overlapping lines). The sharpest prior, $\sigma_p^2 = 5$, has the lowest error for estimations around the mean of the prior.
